# Acceptability, User Satisfaction, and Feasibility of an App-Based Support Service During the COVID-19 Pandemic in a Psychiatric Outpatient Setting: Prospective Longitudinal Observational Study

**DOI:** 10.2196/60461

**Published:** 2024-12-04

**Authors:** Konstanze Golsong, Luisa Kaufmann, Sabrina Baldofski, Elisabeth Kohls, Christine Rummel-Kluge

**Affiliations:** 1 Department of Psychiatry and Psychotherapy Medical Faculty Leipzig University Leipzig Germany; 2 Department of Psychiatry and Psychotherapy University of Leipzig Medical Center Leipzig Germany

**Keywords:** mental health, eHealth, app, health care, app-based support, psychiatric symptoms, mobile phone, COVID-19

## Abstract

**Background:**

Patients with mental disorders often have difficulties maintaining a daily routine, which can lead to exacerbated symptoms. It is known that apps can help manage mental health in a low-threshold way and can be used in therapeutic settings to complement existing therapies.

**Objective:**

The aim of this study was to evaluate the acceptability, usability, and feasibility of an app-based support service specifically developed for outpatients with severe mental disorders in addition to regular face-to-face therapy during the COVID-19 pandemic.

**Methods:**

Patients in a psychiatric outpatient department at a German university hospital were invited to use an app-based support service designed transdiagnostically for mental disorders for 4 weeks. The app included 7 relaxation modules, consisting of video, audio, and psychoeducational text; ecological momentary assessment–like questionnaires on daily mood answered via a visual smiley-face scale; and an activity button to record and encourage daily activities. Standardized questionnaires at baseline (T0; preintervention time point) and after 4 weeks (T2; postintervention time point) were analyzed. Feedback via the smiley-face scale was provided after using the app components (T1; during the intervention). Measures included depressive symptoms, quality of life, treatment credibility and expectancy, and satisfaction. Furthermore, participation rates, use of app modules and the activity button, and daily mood and the provided feedback were analyzed (T2).

**Results:**

In total, 57 patients participated in the study, and the data of 38 (67%) were analyzed; 17 (30%) dropped out. Satisfaction with the app was high, with 53% (30/57) of the participants stating being rather satisfied or satisfied. Furthermore, 79% (30/38) of completers stated they would be more likely or were definitely likely to use an app-based support service again and recommend it. Feasibility and acceptability were high, with nearly half (18/38, 47%) of the completers trying relaxation modules and 71% (27/38) regularly responding to the ecological momentary assessment–like questionnaire between 15 and 28 times (mean 19.91, SD 7.57 times). The activity button was used on average 12 (SD 15.72) times per completer, and 58% (22/38) felt “definitely” or “rather” encouraged to perform the corresponding activities. Depressive symptomatology improved significantly at the postintervention time point (*P*=.02). Quality of life showed a nonsignificant increase in the physical, psychological, and social domains (*P*=.59, *P*=.06, and *P*=.42, respectively) and a significant improvement in the environment domain (*P*=.004). Treatment credibility and expectancy scores were moderate and significantly decreased at T2 (*P*=.02 and *P*<.001, respectively). Posttreatment expectancy scores were negatively associated with posttreatment depressive symptomatology (*r*=–0.36; *P*=.03).

**Conclusions:**

App-based programs seem to be an accessible tool for stabilizing patients with severe mental disorders, supporting them in maintaining a daily routine, complementing existing face-to-face treatments, and overall helping respond to challenging situations such as the COVID-19 pandemic.

## Introduction

### Background

An increasing number of studies illustrate the interest in and benefits of apps in the mental health sector as a support for traditional therapy or stand-alone self-management, especially for depression and anxiety [1]. Apps can be helpful for people in managing their own mental health in a low-threshold way [2,3] and can be used in therapeutic settings to supplement already effective therapy methods, even in acute psychiatric care hospitals [4].

Mobile apps are also on the rise in the mental health research field [1,5]. In 2016, a total of 259,000 apps in the mobile health (mHealth) care sector were listed on major app stores [6], with one-third of disease-specific apps being mental health apps [7]. In 2021, over 350,000 digital health apps were reportedly available in app stores [8]. These numbers reveal the great growth in this area and that many people see the potential of apps as a support in the mental health setting.

Smartphone use is widespread among the population, and >90% of patients in the psychiatric outpatient department at University Hospital Leipzig, Germany, report using a smartphone and the internet [9]. People with mental illnesses use the internet for mental health reasons, for example, to obtain information about mental illness or medication [10]. There is great interest in web-based services for self-help and prevention [9]. Against this background, as well as the frequent private smartphone use in everyday life, it is obvious that smartphones represent an excellent opportunity to offer low-threshold support services in the form of app-based interventions. Thus, app-based interventions also have a decisive advantage over other internet-based offerings. Their potential increases even further against the backdrop of the COVID-19 pandemic and associated limitations in individual and group face-to-face therapy, especially considering the fact that digital interventions were shown to mitigate the negative psychological effects of the pandemic [11].

Ecological momentary assessment (EMA) is a useful tool to monitor the behavior and experiences of an individual repeatedly in real time in their natural environment. It helps capture the dynamic of experiences over a period, thus helping strengthen the understanding of interactions between individuals and their environment [12,13]. In this study, EMA also served as feedback for users themselves to better monitor their mood and activities.

Many of the apps established to date are disease specific and tailored toward people with a specific diagnosis [14]. There are, among others, apps for people with depression [15,16], anxiety [15,16], and obsessive-compulsive disorder [17], which offer the advantage that the content can be specifically adapted to each diagnosis. However, many psychiatric patients present psychiatric comorbidity and have multiple mental disorders [18,19]. Due to this complexity of mental health disorders, transdiagnostic approaches can be found in different areas of mental health research [20,21]. As the goal of this study was to create an app that could potentially be used by all patients in a psychiatric outpatient department, the challenge was to create content that was broad enough to appeal to and be helpful for all patients regardless of their diagnosis or diagnoses but at the same time specific enough to offer optimal support in the psychiatric context and have a positive effect on the patients’ quality of life (QOL), as well as potentially being able to support achieving stability of symptoms in patients who are severely ill.

### Objectives

The aim of this study was to evaluate the feasibility, acceptability, and user satisfaction of an app-based support service for patients receiving treatment for any mental disorder in a psychiatric outpatient department during the COVID-19 pandemic. As other therapeutic offers were limited at that time, support was particularly necessary. We hypothesized that the implementation of an app-based support service would be feasible and that acceptability and satisfaction would be high in that patient group.

## Methods

### Study Design

This prospective longitudinal observational study using an EMA approach evaluated a mental health app that was developed to support psychiatric patients in an outpatient department in Germany in their everyday lives via guided relaxation exercises, short daily EMA-like questionnaires, and an activity button to support patients in planning and monitoring daily activities as well as encourage them to perform the activities. The study population was as broad as possible and not chosen based on diagnosis-specific criteria.

### Ethical Considerations

This study received approval from the ethics committee of the Faculty of Medicine of the University of Leipzig (558/21-ek; December 20, 2021) and was registered in the German Clinical Trials Register (DRKS0027536). Informed consent was given by the participants who wanted to take part in the study, and they could opt out from the study at any time via either deleting their data themselves or informing the work group via telephone or e-mail. Any data collected were deidentified by assigning participants a code randomly generated by the m-path program. No compensation was given for participation in the study.

### Focus Group

To best adapt an app to the needs of users, it is necessary to involve different groups in the development process [22], which means that, among other things, user involvement is necessary. Therefore, in the context of patient-centered research, a focus group session was held at the beginning of the development process of the app and before the start of the study (December 2021) including 2 female patients from the outpatient department, the head of the outpatient department (CR-K), the deputy head of the working group (EK), and the 2 PhD students who were conducting the study (KG and LK). For pandemic safety reasons, the meeting took place online. During the meeting, the expectations and desires of the potential target group were discussed. These suggestions were considered and incorporated during the development of the app.

### Participants and Recruitment

**Figure 1:**
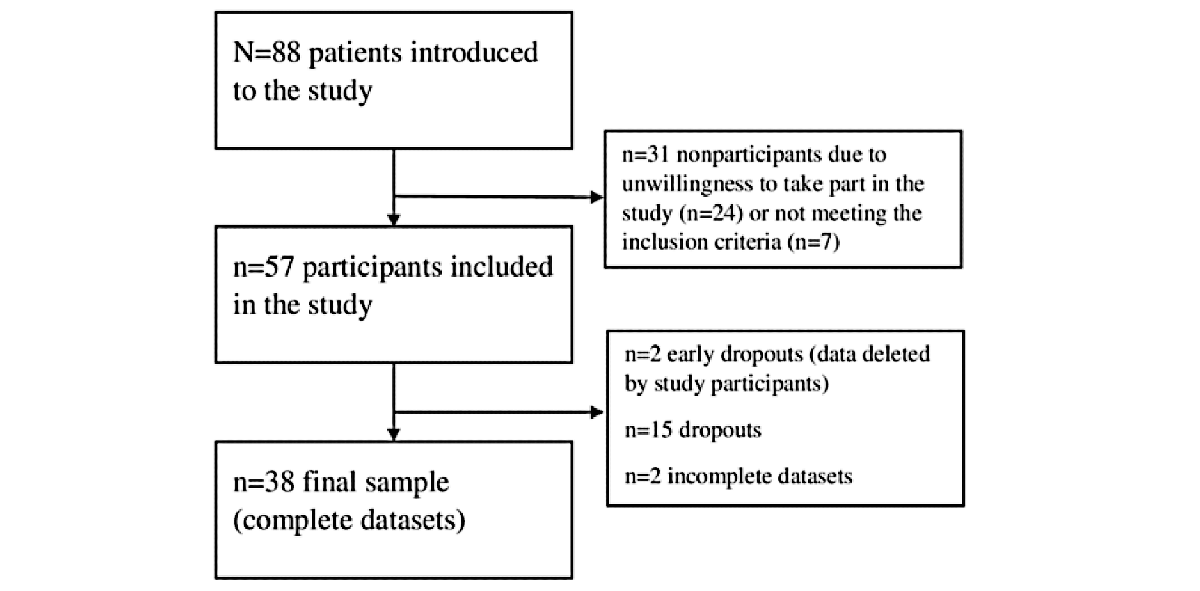
Flowchart of the data collection process.

Recruitment of study participants took place after the completion of app development and content implementation (for details, see the App Intervention section) in February 2022 and March 2022. The study was presented to all patients having face-to-face individual or group therapy appointments at the outpatient department during the recruitment period (88 patients in total; Figure 1). Patients were informed about the study before the start or at the end of an individual or group therapy session. If a patient was interested in study participation and provided written informed consent, an appointment was then made to install the app after the therapy session.

Patients with all mental health disorders were approached to ensure the broadest possible study population. Inclusion criteria were being aged ≥18 years, currently being treated in the psychiatric outpatient department, having sufficient German language skills, having appropriate eyesight and reading abilities, owning a smartphone that supported downloading the app and adequate knowledge of the smartphone, having internet access, and being able to complete the questionnaires independently. Exclusion criteria were pregnancy and breastfeeding. After participants provided written informed consent for taking part in the study and signed the data protection regulations, a handout was distributed with study information as well as an installation guide and manual for app installation and use. Phone numbers were noted for a follow-up call after 1 week to determine whether any problems or questions arose during app use.

Following this procedure, 88 participants were introduced to the study; 57 (65%) were included in the study and started to use the app. Of these 57 participants, 2 (4%) deleted their datasets shortly after study start, 15 (26%) were dropouts due to app nonuse for ≥1 week, and 2 (4%) did not complete the final questionnaire. In total, 38 participants had complete datasets and were included in the final sample (Figure 1).

### Definition of Completers and Dropouts

Completers (40/57, 70%) were defined as everyone who used any app content at least once per week during the 4-week intervention period. Dropouts (17/57, 30%) were defined as all participants who provided written informed consent and filled out the baseline questionnaire (T0; preintervention time point) but who did not use the app for a full week during the 4-week intervention period or who informed the research team by phone or email that they did not want to continue taking part in the study. An anonymous paper-and-pencil questionnaire was used to obtain the reasons for nonparticipation from all 35% (31/88) of the patients who rejected participation in the study and did not provide written informed consent; the results of this questionnaire as well as the evaluation of the dropouts will be published in a subsequent work [23].

### App Intervention

For this study, a mental health app was developed to support psychiatric patients in a psychiatric outpatient department in Germany in their everyday lives and monitor participants’ mental state and behavior while giving them the possibility of self-monitoring. This was done via videos and audios as well as psychoeducational texts to perform relaxation and mindfulness exercises or learn more about daily situations, such as scheduling activities, developing structure, or improving sleep. In addition, short daily questionnaires using an EMA-like approach were part of the app as this has been shown to be a helpful tool especially to monitor depressive symptomatology [24]. Furthermore, an activity button was implemented to make patients aware of their planned daily activities and later indicate whether they had performed these planned activities. If no activities were planned, examples of possible activities were given to create inspiration and encourage participants to perform an activity. Participants were also suggested to use the various relaxation and mindfulness modules as behavioral activation is beneficial in treating a number of mental illnesses [25,26].

The app’s primary goal was to stabilize and monitor a participant’s mental state and behavior while giving them the possibility of self-monitoring. It was built using the m-Path system, which was developed at Katholieke Universiteit Leuven, Belgium [27]. m-Path was originally built as a platform for smartphone-based EMA and smartphone-based intervention for clinical practice and behavioral research, but it also offers the possibility to design stand-alone (self-help) applets including instructions or multimedia content [27]. It allows the creation of an app tailored to individual needs, aims, and requirements within a predefined modular system.

m-Path proved to be a useful platform due to several aspects. First, the app that can be created is usable for both Android and iOS [27]; second, no knowledge of programming languages is required to create app content [27]; third, the platform offers an award system to increase user compliance [27]; fourth, end-to-end encryption is used for phone-server communication, ensuring data safety; fifth, participants can independently delete their data if needed; and, finally, participants do not have to provide personal data, such as name, email address, or phone number, to sign up and use the app, making m-Path a secure platform [27].

**Figure 2:**
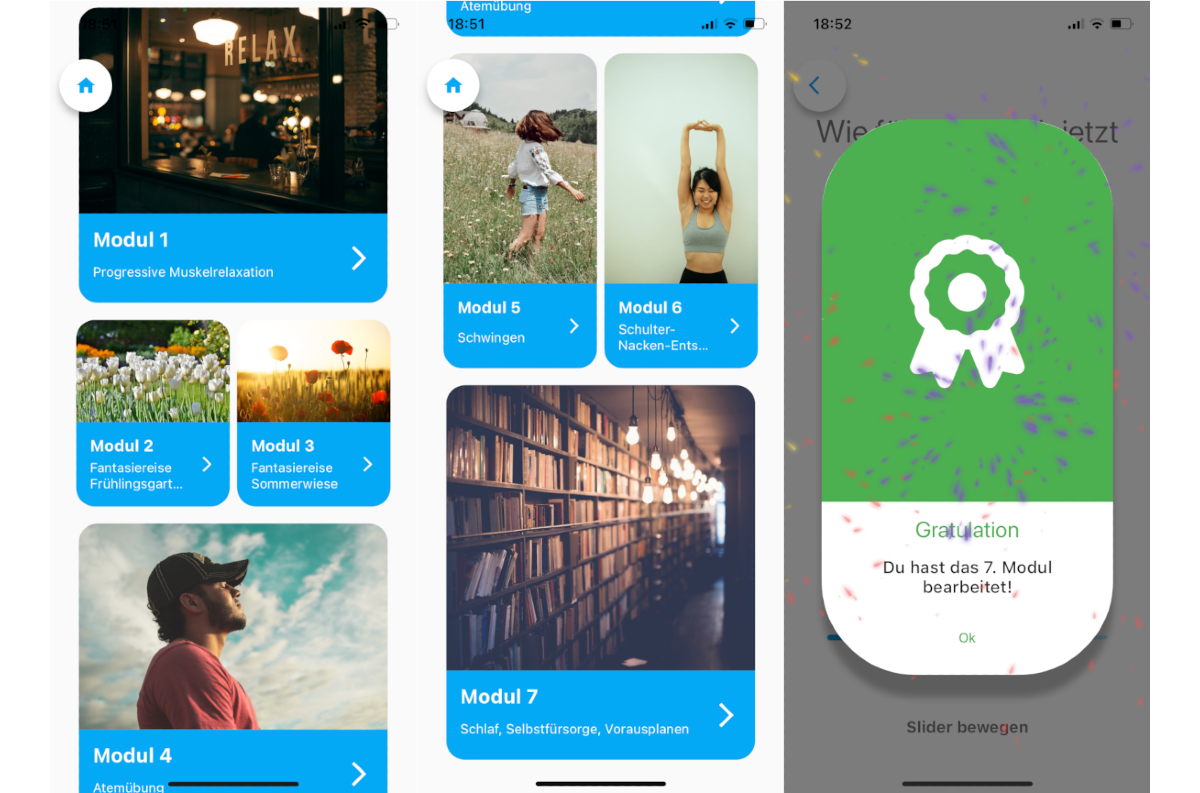
Screen interface of the app modules and award following module use.

All content for the app used in this study was developed, created, and implemented by the authors after previous extensive literature research and conducting a focus group. Seven different modules were developed so that participants could choose between videos, audios, and psychoeducational texts to perform relaxation and mindfulness exercises or learn more about common situations, such as scheduling activities, developing structure, or improving their sleep: (1) module 1—Jacobson’s progressive muscle relaxation (audio); (2) module 2—fantasy journey spring garden (audio); (3) module 3—fantasy journey summer meadow (audio); (4) module 4—breathing exercise (audio); (5) module 5—movement exercise (video); (6) module 6—shoulder-neck relaxation (video); and (7) module 7—psychoeducational texts on sleep, self-care, and planning ahead. The goal was to provide content that would appeal to and be helpful to patients with different diagnoses. Screenshots of the layout and structure of the app can be found in Figure 2. After using a module, patients were asked how they liked it on a smiley-face scale (0-100).

**Figure 3:**
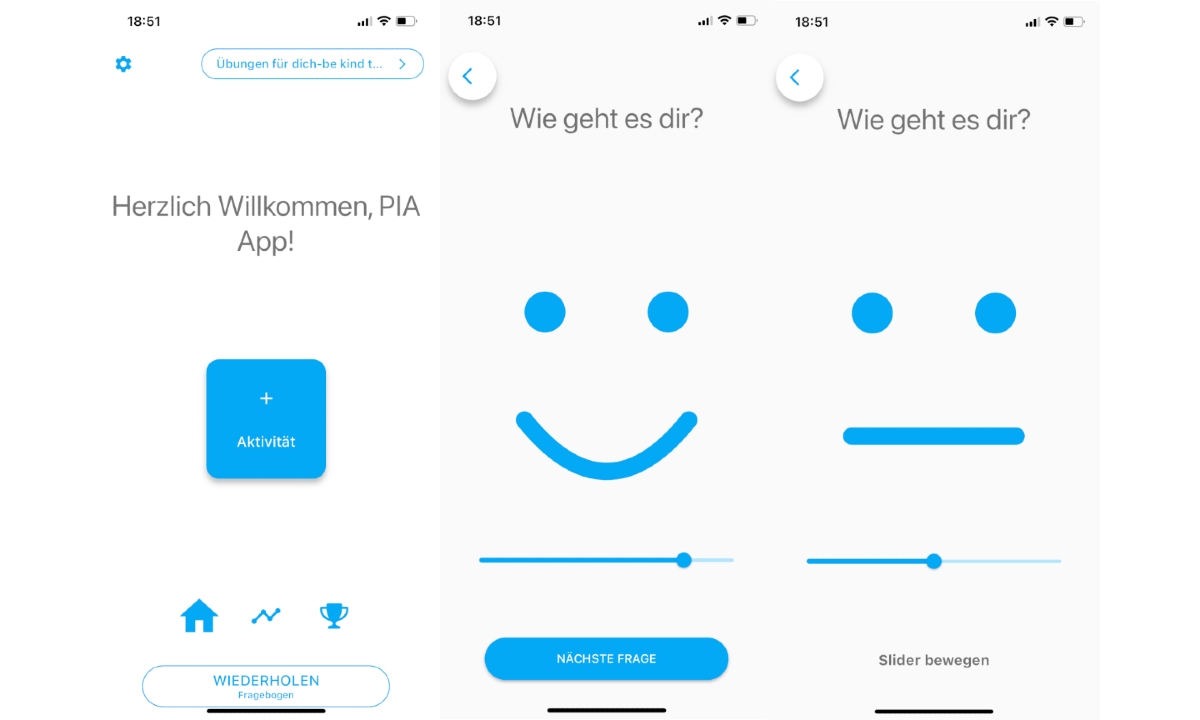
App home screen and visual smiley-face scale.

In addition to the modules, the app contained regular EMA-like questionnaires assessing the participants’ mood. Participants were sent a short questionnaire via the app 3 times a day, which they were reminded of via a push notification. By means of this questionnaire, their current mood was assessed using a smiley-face scale (Figure 3). The participants were able to complete each of the 3 questionnaires (morning questionnaire: 6 AM-11:59 AM; daytime questionnaire: noon-5:59 PM; evening questionnaire: 6 PM-11:59 PM) a maximum of 28 times over the study duration (once per day, 7 times per week for the 4-week study duration).

An additional feature was the possibility to record activities via a button on the home screen, where participants could enter their planned activities for the day and later indicate whether they had performed these planned activities. If the participants had indicated via the app that they had not planned any activity, various options of potential activities were provided to create inspiration and motivation to perform an activity. Participants were also suggested to use the various relaxation and mindfulness modules for behavioral activation, which has been proven helpful for various mental illnesses [25,26].

The different relaxation and mindfulness modules, as well as the activity button, could be used indefinitely. After each activity button use if a planned activity was performed and after each module use, participants received awards (trophies, badges, or fireworks) as gamification. The collected awards could be viewed on the app. This procedure was intended for motivating the participants to keep using the app and see their progress as gamified app features have the ability to encourage positive health behaviors [28].

The use of the app was limited to a period of 4 weeks. The baseline questionnaire (T0) was activated on the smartphone immediately before app use, and the postintervention questionnaire (T2) was activated after study completion. The study administrators could directly see the results of the questionnaires on the web-based dashboard, which was important to be able to react directly to possible suicidal thoughts (item 9 of the Patient Health Questionnaire–9 [PHQ-9] specifically asks about suicidal thoughts or self-injurious behavior; further information can be found in the Safety Procedures section). App use was only possible after the baseline questionnaire (T0) was completed. A reminder to complete the postintervention questionnaire (T2) was sent via a push notification on the smartphone, and if necessary, the participant was reminded via a phone call.

One benefit of m-Path is that participants could obtain an overview of when they used which app components by means of a graphical visualization on the app itself, and based on their answers to the EMA-like questionnaires, they were shown a curve of how their mood varied over the period of the study [27].

### Safety Procedures

The baseline (T0) and postintervention (T2) questionnaires specifically asked about suicidal thoughts or self-injurious behavior (item 9 of the PHQ-9). The research team was able to view the responses to these questionnaires. The participants received an automated warning message if they indicated to have suicidal thoughts on ≥1 day within the previous 2 weeks. In this case, the standard operating procedure (SOP) from the psychiatric outpatient department was carried out. At T0, this included a clinical face-to-face assessment of the suicidal thoughts and a consultation with the physician on duty if necessary. At T2, the participants again received a warning message, were contacted by telephone, and received an assessment of the suicidal thoughts; in an emergency, the rescue service was contacted. In total, 25% (14/57) of the participants as indicated in the baseline questionnaire (T0) and 19% (11/57) of the participants as indicated in the postintervention questionnaire (T2) generated an SOP regarding suicidal thoughts.

### Measures

#### Overview

Outcomes were measured through self-report questionnaires via the app. Assessments took place at baseline, immediately after informed consent for study participation was given and before the app could be used (T0; preintervention time point); after every in-app module use (T1; during the intervention); and at the end of the intervention or app use after 4 weeks (T2; postintervention time point). The preintervention questionnaire (T0) asked participants about basic sociodemographic characteristics, including employment status, living situation, parenthood, marital status, and psychological distress due to the COVID-19 pandemic.

#### User Satisfaction

At the postintervention evaluation (T2), the participants were asked to fill in an adapted version of the German version of the Client Satisfaction Questionnaire–8 (ZUF-8) [29,30] to measure their satisfaction with the general app use and specific app content.

As a variety of app content (videos and audios) was evaluated, the wording of the individual items of the ZUF-8 was adjusted accordingly. In total, 8 items were assigned to “audio” and “video,” of which 5 items overlapped as their wording was not content specific but general (ie, “How would you describe the operation of the app?”). The content-specific questions were slightly adjusted (ie, “How would you rate the quality of the videos?” and “How would you rate the quality of the audios?”). Thus, there were ultimately 2 ZUF-8 results, one for video content and one for audio content.

All items were measured on a 4-point Likert scale from 1 (low satisfaction) to 4 (highest satisfaction). The participants had to answer whether the videos and audios helped them relax or had a positive impact on their psychological well-being, whether they would recommend the app with these videos and audios to a friend, and whether they would use a similar app approach if they were in need of help again.

The total sum scores ranged from 8 to 32, with higher scores indicating higher satisfaction. This questionnaire was chosen because of its high reliability, although the internal consistency was only sufficient [30]. In previous studies, a ZUF-8 score of ≥20 (out of 32) was assumed to be acceptable, so this cutoff was also used in this study [31-33].

Furthermore, three additional questions independent of the ZUF-8 were asked to evaluate the activity button use and satisfaction with the EMA-like questionnaire: (1) “Did the activity button help you to remember planned activities?” (2) “Did the activity button encourage you to perform activities?” (3) “Did you find the three daily questionnaires (the EMA-like questionnaires) appropriate in terms of frequency?”

Finally, at the completion of the study, participants had the opportunity to provide feedback as free text within the app; in addition, all 57 study participants (dropouts and completers) were asked for feedback by telephone as well as what school grade (with 1 as the best and 6 as the worst) they would give the app-based support service.

#### Depressive Symptoms

To assess depressive symptomatology at baseline (T0; preintervention time point) and for comparison at the end of the intervention (T2; postintervention time point), the PHQ-9 [34] was administered. This questionnaire includes 9 items on a 4-point Likert scale from 0 (*not at all*) to 3 (*nearly every day*). If item 9 (suicidal thoughts and self-injurious behavior) of the PHQ-9 was ≥1, the SOP from the psychiatric outpatient department was carried out (see the Safety Procedures section for more information). Higher scores indicate more severe depressive symptoms, with the total sum score ranging from 0 to 27. Levels of severity were used to categorize the scores, with scores of ≥10 indicating clinically relevant depressive symptoms. Numerous studies have shown excellent validity for the PHQ-9 [35]. A more recent meta-analysis showed that the PHQ-9 sensitivity was higher than in earlier conventional meta-analyses compared to semistructured diagnostic interviews. A cutoff score of ≥10 was shown to maximize combined sensitivity and specificity [36]. The internal consistency and test-retest reliability of the PHQ-9 provided via smartphone has been proven to be high [37].

#### QOL Measurement

To measure the participants’ QOL, the World Health Organization Quality of Life assessment (WHOQOL-BREF) was used [38] and administered at baseline and the postintervention time point. This questionnaire consists of 26 items that ask about the life domains of physical health, psychological health, social relations, and environment on a 5-point Likert scale from 1 (*not at all*) to 5 (*extremely*). For each of these life domains, domain scores ranging from 0 to 100 were calculated. A higher score indicates a higher QOL. The WHOQOL-BREF has demonstrated strong internal consistency, item-total correlations, discriminant validity, and construct validity [39].

#### Treatment Expectancy and Credibility

The Credibility/Expectancy Questionnaire (CEQ) was used to measure treatment credibility [40]. It was administered at baseline and at the end of the intervention at the postintervention time point. A German translation of the CEQ was used that had been developed in the working group in a previous project [41]. The wording of the CEQ was slightly adapted to the app format. The questionnaire consists of a credibility factor (3 items) and an expectancy factor (3 items). The 3 items of the credibility factor and item 2 of the expectancy factor are measured on a scale from 1 to 9. However, items 1 and 3 of the expectancy factor use an 11-point scale from 0% to 100%. This 11-point scale was transformed into a 9-point scale so that the total sum scores for both the credibility and expectancy factors ranged between 3 and 27. A higher total score indicated a higher credibility or expectancy. High internal consistency and good test-retest reliability have been found for the CEQ [40].

### Pandemic Situation in Germany During the Study Period

At the time when this study was conducted (February 2022-March 2022), the German population faced restrictions related to the COVID-19 Omicron wave to protect against infection; there were >120,000 COVID-19 deaths in Germany, and the 7-day incidence was >1100. The population faced many changes in regulations within a short time frame. In the psychiatric outpatient department where the study was conducted, individual and group therapies with the wearing of a medical mask were possible [42].

### Operationalization of Acceptability, Feasibility, and Satisfaction

The 3 parts on which the main study hypothesis was based were acceptability, feasibility, and user satisfaction. The intervention was considered to be of significant feasibility if (1) it was carried out as recommended by most participants (ie, the app was used at least once per week during the 4-week study period), (2) every participant could use the app independently, (3) most participants considered app use to be rather or very easy, and (4) the quality of both the video and the audio files was rated as rather good or good.

In addition, the intervention was considered satisfactory if (1) a mean sum score of at least 20 was obtained on the modified ZUF-8, (2) most participants indicated that they would rather or definitely recommend the app to a friend, (3) most participants indicated that they were more likely or definitely likely to use an app-based support service in the future if they needed help again, and (4) most participants rated the app’s overall usability as rather satisfactory or satisfactory.

An intervention must be acceptable for it to be successfully implemented [43]. It was previously established from earlier studies that, if an intervention is considered acceptable, patients are more likely to follow treatment recommendations and benefit from improved clinical results [44,45]. Therefore, if the intervention in our study is satisfactory and feasible in accordance with the aforementioned specifications, we also consider it acceptable.

### Statistical Analysis

First, descriptive analyses of sociodemographic variables and psychological distress due to the COVID-19 pandemic were conducted. Second, the extent of use of the various app components (modules, EMA-like questionnaires via a smiley-face scale, and activity button) by study participants was evaluated. Descriptive analyses further included depressive symptoms (PHQ-9), QOL (WHOQOL-BREF), treatment expectancy and credibility (CEQ), and participant satisfaction (ZUF-8). Particular attention was paid to the descriptive analysis of item 9 (suicidal thoughts and self-injurious behavior) of the PHQ-9 and whether there was a change in the frequency at which the SOP was carried out at T0 and T2.

Next, potential differences among depressive symptoms (PHQ-9), QOL (WHOQOL-BREF), and credibility and expectancy scores (CEQ) between baseline and the postintervention time point were analyzed. Before testing for potential differences, normal distribution was assessed using the Shapiro-Wilks test. As PHQ-9 scores were normally distributed, a 2-tailed *t* test for paired samples was used. The WHOQOL-BREF at baseline and the postintervention time point was normally distributed in all domains except for domain 4 (environment) at the posttreatment time point, so potential differences in domains 1 to 3 were assessed using *t* tests for paired samples and domain 4 was analyzed using the Wilcoxon test (nonparametric paired groups). The credibility score on the CEQ at baseline and the postintervention time point was not normally distributed, so potential differences were assessed using the Wilcoxon test (nonparametric paired groups) and the expectancy scores were analyzed using a *t* test for paired samples.

In addition, correlation analyses between treatment credibility and expectancy at baseline and the postintervention time point (CEQ) and between baseline and postintervention depressive symptoms (PHQ-9) and QOL (WHOQOL-BREF) were conducted using the Pearson correlation coefficient.

Finally, the mean values of the EMA-like questionnaires were determined and presented graphically in the form of a mood analysis for the duration of the study.

All statistical testing was 2-tailed at a level of α=.05 except for the WHOQOL-BREF, where a Bonferroni correction (α/4) was used to account for multiple testing. Analyses were performed using SPSS (version 27.0; IBM Corp).

## Results

### Sample Characteristics

Overall, 57 participants with ages ranging from 18 to 68 years (mean 37.21, SD 13.55 y) were included in the study (Table 1). The sample’s gender distribution was unequal, with almost three-quarters (42/57, 74%) being women and slightly more than one-quarter (15/57, 26%) being men.

**Table 1:** Sample characteristics of the entire study population and completers.

Variable		Full cohort^a^ (n=57)	Completers (n=38)
Age (y), mean (SD)		37.21 (13.55)	39.45 (14.13)
Women, n (%)		42 (74)	30 (79)
**Relationship status, n** **(%)**			
	Married	15 (27)	12 (32)
	Divorced	4 (7)	3 (7)
	In a relationship	14 (25)	8 (21)
	Single	22 (40)	15 (39)
Living situation with others, n (%)		39 (71)	25 (66)
Children, n (%)		28 (51)	21 (55)
Children living in the household, n (%)		20 (36)	14 (37)
**Employment status, n** **(%)**			
	Employed	17 (31)	12 (32)
	Unemployed	3 (5)	1 (3)
	Retired or unable to work	15 (27)	13 (34)
	In school, training or studying	15 (27)	8 (21)
	On parental leave or housewife or househusband	5 (9)	4 (11)
**Main diagnosis (*ICD-10-CM* ^b^ ), n (%)**			
	Emotionally unstable personality disorder	6 (11)	4 (11)
	Attention-deficit/hyperactivity disorder	9 (16)	4 (11)
	Panic disorder	5 (9)	4 (11)
	Generalized anxiety disorder	4 (7)	3 (8)
	Paranoid schizophrenia	4 (7)	3 (8)
	Recurrent depressive disorder, currently moderate episode	5 (9)	4 (11)
	Recurrent depressive disorder, currently severe episode without psychotic symptoms	4 (7)	2 (5)
	Other	20 (35)	14 (37)

^a^Calculation of percentages from valid cases. Missing data were n=2 for relationship status, living situation, children, children living in the household, employment status.

^b^ICD-10-CM: International Classification of Diseases, 10th Revision, Clinical Modification.

A total of 38 participants were included in the final sample. Of these 38 participants, 15 (39%) were single, 20 (53%) were married or in a relationship, and 3 (8%) were divorced. Most (25/38, 66%) lived with other people (eg, partner, children, or roommates). A little over half (21/38, 55%) of the participants had children, whereas just over one-third (14/38, 37%) lived with children in the household. Almost one-third (12/38, 32%) of the participants were currently employed, whereas 3% (1/38) of the participants were unemployed, and 13% (5/38) were unable to work. There was an equal distribution of individuals who were either retired (8/38, 21%) or enrolled in school, training, or study (8/38, 21%). Concerning their diagnosis, 11% (4/38) had major depression, 11% (4/38) had emotionally unstable personality disorder, 11% (4/38) had attention-deficit/hyperactivity disorder, 11% (4/38) had panic disorder, 8% (3/38) had paranoid schizophrenia, and 42% (16/38) had other mental disorders.

Furthermore, four-fifths of the participants (31/38, 82%) felt somewhat or very distressed due to the pandemic. Over two-thirds (26/38, 69%) of the participants subjectively felt clearly (8/26, 31%) or rather (18/26, 62%) constrained by the COVID-19 containment measures, whereas one-third (12/38, 32%) felt not constrained (3/12, 25%) or rather not constrained (9/12, 75%).

### Participation

#### Module Use

Participants of the full cohort used between none and all 7 modules. Over a third of participants (22/57, 39%) tried 1 or 2 modules. Over one-tenth (7/57, 12%) of the participants used ≥5 of the modules at least once. Almost half (25/57, 44%) of the participants did not try any of the modules. With 30 uses in total across all participants of the full cohort, module 7 (psychoeducational texts on sleep, self-care, and planning ahead) was the most frequently used. It was used once by 30% (17/57) of the participants, 4% (2/57) of the participants used the module 3 times, and it was used 5 and 6 times by 2% (1/57) of the participants in each case. Module 4 (breathing exercise) was the second most used module, with 24 uses in total across all participants. A total of 18% (10/57) of the participants used the module once; 9% (5/57) of the participants used the module between 2 and 4 times. Module 2 (fantasy journey spring garden) had 21 views. In total, 18% (10/57) of the participants used this module once; 7% (4/57) of the participants used this module 2 to 3 times. Module 1 (Jacobson’s progressive muscle relaxation) and module 3 (fantasy journey summer meadow) had a similar use rate, with 17 and 15 views, respectively. Module 1 was accessed once by 14% (8/57) of the participants, and it was used 3 and 6 times by 4% (2/57) of the participants in each case. Module 3 was used between once and 6 times by 11% (6/57) of the participants. The movement exercises, module 5 (movement exercise) and module 6 (shoulder-neck relaxation), were used the least, with 13 and 11 views, respectively.

Completers used between none and all 7 modules. Almost half (18/38, 47%) of the participants tried 1 or 2 modules. Nearly one-fifth (7/38, 18%) of the participants used ≥5 of the modules at least once. A quarter (10/38, 26%) of the participants did not try any of the modules. With 28 uses in total across all participants, module 7 (psychoeducational texts on sleep, self-care, and planning ahead) was the most frequently used. It was used once by 29% (11/38) of the participants, 5% (2/38) of the participants used the module 3 times, and it was used 5 and 6 times by 3% (1/38) of the participants in each case. Module 4 (breathing exercise) was the second most used, with 22 uses in total across all participants. A total of 21% (8/38) of the participants used the module once; 13% (5/38) of the participants used the module between 2 and 4 times. Module 2 (fantasy journey spring garden) had 20 views. In total, 24% (9/38) of the participants used this module once; 11% (4/38) of the participants used this module 2 to 3 times. Module 1 (Jacobson’s progressive muscle relaxation) and module 3 (fantasy journey summer meadow) had a similar use rate, with 16 and 15 views, respectively. Module 1 was accessed once by 18% (7/38) of the participants, and it was used 3 and 6 times by 5% (2/38) of the participants in each case. Module 3 was used between once and 6 times by 16% (6/38) of the participants. The movement exercises, module 5 (movement exercise) and module 6 (shoulder-neck relaxation), were used the least, with 13 and 11 views, respectively.

#### Use of the EMA-Like Questionnaire via a Visual Smiley-Face Scale

Among the full cohort, most (49/55, 89%) answered all 3 EMA-like questionnaires at least once. On average, the questionnaire in the morning was answered 15 (SD 10.1) times, with almost one-third (18/55, 33%) of the participants answering the questionnaire between 22 and 28 times and almost one-fifth (10/55, 18%) answering it as often as 15 to 21 times (Table 2). The EMA-like questionnaire during the day was answered an average of 15 (SD 9.9) times, with over one-third (19/55, 35%) of the participants answering it 22 to 28 times and just under one-fifth answering it between 15 and 21 times (9/55, 16%) and between 8 and 14 times (10/55 18%; Table 2). The EMA-like questionnaire in the evening achieved similar values. On average, it was also answered 15 (SD 10.0) times, and a total of 40% (22/55) of the participants answered it between 22 and 28 times (Table 2).

**Figure 4:**
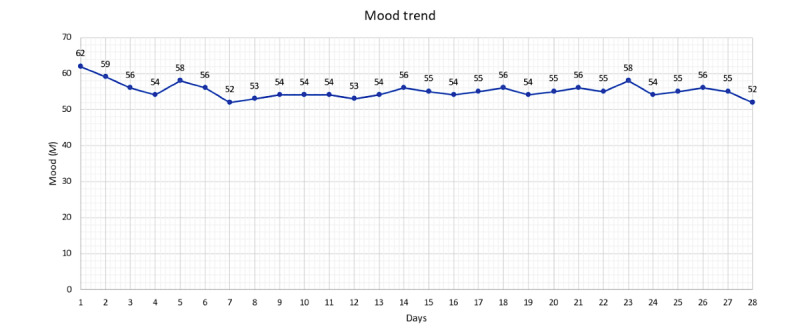
Mean mood trend for completers of the 4-week study duration via ecological momentary assessment-like questionnaires.

Of the completers, all except for 1 study participant (37/38, 97%) answered all 3 EMA-like questionnaires at least once. On average, the questionnaire in the morning was answered 20 (SD 7.4) times, with almost half (18/38, 47%) of the participants answering the questionnaire between 22 and 28 times and just over a quarter (10/38, 26%) answering it as often as 15 to 21 times (Table 3). The EMA-like questionnaire during the day was answered an average of 19 (SD 7.5) times, with half (19/38, 50%) of the participants answering it 22 to 28 times and approximately one-fifth answering it between 15 and 21 times (8/38, 21%) and between 8 and 14 times (8/38, 21%; Table 3). The EMA-like questionnaire in the evening achieved similar values. On average, it was answered 20 (SD 7.8) times, and a total of 58% (22/38) of the participants answered it between 22 and 28 times (Table 3). Figure 4 provides a graphical representation of the mean mood trend for the duration of the study determined using the EMA-like questionnaires (the values have been rounded to whole numbers for better presentation). When asked whether the EMA-like questionnaires were appropriate in terms of frequency, 84% (32/38) of the participants answered *yes* or *rather yes*. A minority of only 16% (6/38) of the participants found the frequency rather not or not appropriate.

**Table 2:** Responses to the ecological momentary assessment (EMA)–like questionnaires in the sample of the full cohort (n=55).

Overall number of times the daily EMA-like questionnaires were answered during the 4-week study^a^	Morning questionnaire	Daytime questionnaire	Evening questionnaire
0-7, n (%)	16 (29)	15 (27)	18 (33)
8-14, n (%)	11 (20)	12 (22)	10 (18)
15-21, n (%)	10 (18)	9 (16)	5 (9)
22-28, n (%)	18 (33)	19 (35)	22 (40)
Total, mean (SD)	15.15 (10.08)	14.67 (9.87)	14.00 (10.04)

^a^Possible maximum of 28 times per questionnaire (morning, daytime, and evening) over the study duration (once per day, 7 times per week for the 4-week study duration).

**Table 3:** Responses to the ecological momentary assessment (EMA)–like questionnaires in the sample of completers (n=38).

Overall number of times the daily EMA-like questionnaires were answered during the 4-week study^a^	Morning questionnaire	Daytime questionnaire	Evening questionnaire
0-7, n (%)	2 (5)	3 (8)	3 (8)
8-14, n (%)	8 (21)	8 (21)	8 (21)
15-21, n (%)	10 (26)	8 (21)	5 (13)
22-28, n (%)	18 (47)	19 (50)	22 (58)
Total, mean (SD)	20.26 (7.4)	19.39 (7.5)	20.08 (7.8)

^a^Possible maximum of 28 times per questionnaire (morning, daytime, and evening) over the study duration (once per day, 7 times per week for the 4-week study duration).

#### Activity Button Use

The activity button was used to enter a planned activity between 0 and 78 times by the study participants over the study duration. Among those who completed the study, it was used an average of 12 (SD 15.72) times per participant (median 6). Among the full cohort, it was used an average of 10 (SD 13.82) times per participant (median 4). According to the completers, the use of the activity button encouraged them to perform activities. Most of the completers (22/38, 58%) reported that the button definitely (9/22, 41%) or rather (13/22, 59%) encouraged them to perform an activity. For most completers, the button did not serve as a reminder of activities (*no*: 5/38, 13%; *rather no*: 17/38, 45%). There are no data available for the full cohort; due to dropout, the questionnaire that administered these questions was not answered (T2).

### Satisfaction

Among those who completed the study, a mean sum score of 23.0 (SD 4.02) was obtained on the modified ZUF-8 regarding the video content, and a mean sum score of 23.1 (SD 4.23) was obtained on the modified ZUF-8 regarding the audio content. Of those who completed the study, a total of 82% (31/38) were above the cutoff score of 20 regarding the video content, and a total of 84% (32/38) were above the cutoff score of 20 regarding the audio content.

In the posttreatment evaluation, most study participants (30/57, 53%) stated that, overall, they had been rather satisfied (26/30, 87%) or satisfied (4/30, 13%) with the app-based support service. Among completers, more than three-quarters (30/38, 79%) indicated that they were more likely (18/30, 60%) or definitely likely (12/30, 40%) to use an app-based support service in the future if they needed help again. The same number (30/38, 79%) of participants would be more (19/30, 63%) or definitely likely (11/30, 37%) to recommend an app-based support service to a friend if they were in a similar situation.

Of those who completed the study, most (34/38, 89%) classified the use of the app as rather easy (19/34, 56%) or very easy (15/34, 44%). The amount of app use was found to be high. Almost half (18/38, 47%) of those participants used the app daily. Almost a quarter (9/38, 24%) used it >2 times per week. The quality of both the video (35/38, 92%) and audio (34/38, 89%) files was rated as rather good to good.

Regarding the question of whether participants experienced the kind of support they wanted on relaxation or mindfulness through the videos available on the app, the response among completers was balanced between *no*/*rather no* (19/38, 50%) and *rather yes*/*yes* (19/38, 50%). Slightly better results were obtained on the same question asking about the audio files (*no*/*rather no*: 17/38, 45%; *rather yes*/*yes*: 21/38, 55%).

In the posttreatment evaluation, each study participant (dropouts and completers) was asked what school grade (with 1 as the best and 6 as the worst) they would give the app-based support service. Of those who gave an answer (47/57, 82%), most (32/47, 68%) rated the app as excellent (2/47, 4%) or good (30/47, 64%). Just under a third (15/47, 32%) rated the app as satisfactory (13/47, 28%) or sufficient (2/47, 4%). The overall mean school grade was 2.32 (SD 0.63).

After using a module, users were asked how they liked it and could express their opinion using a smiley-face scale (0-100). As most participants, when they used a module, did so only once, the following results refer to the ratings after the first use. Module 1 was given scores from 49 to 100 and achieved the best rating, with a mean of 68.78 (SD 17.8). Modules 3, 5, 4, and 6 received similar ratings, from 46 to 88 (mean 65.17, SD 16.73), 53 to 79 (mean 65.2, SD 9.3), 49 to 82 (mean 62.38, SD 9.64), and 32 to 77 (mean 60.1, SD 13.1), respectively. Modules 7 and 2 performed the worst, with scores of 51 to 85 (mean 55.87, SD 44.2) and 35 to 83 (mean 50.62, SD 46.8), respectively.

### Depressive Symptoms

PHQ-9 scores at baseline and the postintervention time point revealed an average of moderate depressive symptoms in this population, and a mean decrease of 1.5 (T0: mean 13.6, SD 5.7; T2: mean 12.1, SD 5.7; Table 4) on the PHQ-9 sum score was found. Most of the participants (T0: 31/38, 82%; T2: 24/38, 63%) showed moderate to severe symptoms. Depressive symptoms were significantly lower at the end of the intervention than at baseline (t_37_=2.40; *P*=.02; *d*=0.39). Regarding item 9 of the PHQ-9 (suicidal thoughts and self-injurious behavior), the frequency of performing the SOP between baseline and the postintervention time point showed a slight, nonsignificant decrease (T0: 14/38, 37%; T2: 11/38, 29%; *P*=.26). Of the 11 suicide alerts at T2, a total of 9 (82%) were triggered by the same participants as in T0; of these 9 participants, 5 (56%) remained stable at the same level (n=2, 40% remained stable at the level of “on single days”; n=2, 40% remained stable at the level of “on more than half of the days”; and n=1, 20% remained stable at the level of “almost every day”). In the case of the 18% (2/11) of the participants who triggered alerts for the first time at T2, the level increased from “no suicidal thoughts” to “on single days.” For the 21% (3/14) of the participants who no longer triggered suicide alerts, the level dropped from “on single days” to “no suicidal thoughts.”

**Table 4:** Results of assessments of depressive symptoms, quality of life, treatment credibility and expectancy, and satisfaction at baseline (T0) and the posttreatment time point (T2; n=38).

Variable			T0		T2		*P* value^a^
**Depressive symptoms (PHQ-9^b^ sum score)**							.021
	0-4, n (%)	3 (8)		3 (8)			
	5-9, n (%)	4 (11)		11 (29)			
	10-14, n (%)	16 (42)		14 (37)			
	15-19, n (%)	9 (24)		3 (8)			
	20-27, n (%)	6 (16)		7 (18)			
	Sum score, mean (SD)	13.61 (5.7)		12.08 (5.7)			
**Quality of life (WHOQOL-BREF^c^ score), median (IQR)**							
	Physical domain	48.21 (35.71-67.86)		51.79 (39.29-71.43)		.592	
	Psychological domain	45.83 (29.17-54.17)		47.92 (32.29-58.33)		.060	
	Social domain	54.17 (33.33-66.67)		58.33 (41.67-75.00)		.423	
	Environment domain	65.63 (53.13-75.78)		67.19 (56.25-87.50)		.004	
**Treatment credibility and expectancy (CEQ^d^ score), mean (SD)**							
	Credibility factor	18.03 (3.42)		16.58 (3.69)		.015	
	Expectancy factor	14.26 (4.58)		11.66 (5.25)		<.001	

^a^Bonferroni-adjusted *P* values for quality of life (abbreviated World Health Organization Quality of Life assessment score).

^b^PHQ-9: Patient Health Questionnaire–9.

^c^WHOQOL-BREF: abbreviated World Health Organization Quality of Life assessment.

^d^CEQ: Credibility/Expectancy Questionnaire.

### QOL Results

At baseline, the lowest scores were found for the domain of psychological health, and the highest scores were found for the domain of environmental QOL. Overall, the baseline WHOQOL-BREF score revealed a medium level of QOL within the study population. At the posttreatment time point (T2), scores were slightly higher. In addition, in this case, the lowest score was found for psychological health, and the highest score was found for environmental QOL. Physical health and social relations improved slightly. There was no statistically significant difference in the QOL scores on WHOQOL-BREF domains 1 to 3 (physical quality, *P*=.592; psychological quality, *P*=.060; social quality, *P*=.423; Table 4). However, when considering the Bonferroni correction, QOL in relation to domain 4, environmental quality, was still significantly higher after the app intervention (environmental quality, *P*=.004; Table 4).

### Treatment Credibility and Expectancy

The baseline questionnaire revealed medium-high credibility values and moderate expectancy values. The posttreatment survey showed a significant decrease in credibility and expectancy values (Table 4).

A correlation analysis showed a significant negative correlation between the pretreatment expectancy factor of the CEQ and the posttreatment PHQ-9 sum score (*r*=–0.344; *P*=.03). In addition, a significant positive correlation between the pretreatment credibility factor and the posttreatment environmental QOL score (*r*=0.325; *P*=.046) was detected. No significant correlation between the pretreatment credibility factor and the posttreatment PHQ-9 sum score (*r*=–0.101; *P*=.55) as well as all other posttreatment QOL scores (social QOL score: *r*=0.199 and *P*=.23; psychological QOL score: *r*=0.169 and *P*=.31; physical QOL score: *r*=0.168 and *P*=.31) were found. There was also no significant correlation between the pretreatment expectancy factor and all posttreatment QOL scores (environmental QOL score: *r*=0.080 and *P*=.63; social QOL score: *r*=0.169 and *P*=.31; psychological QOL score: *r*=0.273 and *P*=.10; physical QOL score: *r*=–0.112 and *P*=.51).

### Feedback

At the completion of the study, participants had the opportunity to provide feedback as free text within the app; in addition, all 57 study participants (dropouts and completers) were asked for feedback via telephone. In total, 95% (54/57) of the participants provided feedback. The feedback could be categorized into positive, negative, and neutral. The most common subcategories in the positive feedback category were participants liking the app (9/54, 17%), participants liking the brevity of the EMA-like approach (10/54, 19%) and that the EMA-like approach was administered 3 times a day (24/54, 44%), the relaxation exercises being considered helpful (12/54, 26%), and the activity button being considered positive (9/54, 17%). One participant even asked to be allowed to use the app beyond the study period.

The most common subcategories in the negative feedback category were participants not liking the app design (6/54, 11%), a desire for a wider selection of relaxation exercises (8/54, 15%), and a wish for more individualized questions (8/54, 15%).

The most common subcategories in the neutral feedback category were participants not having time to use the relaxation modules (9/54, 17%) or using other relaxation options instead (11/54, 20%).

### Outcome of the Operationalization of Acceptability, Satisfaction, and Feasibility

The app-based support service can be considered feasible regarding the previously defined operationalization of acceptability, feasibility, and satisfaction. The intervention was used at least once by most participants (38/57, 67%) and even daily or >2 times per week by almost half (27/57, 47%) of the participants. Every participant in the final sample (38/38, 100%) used the app independently. While most participants in the full cohort (34/57, 60%) found the app rather or easy to use, among completers, the rate was 89% (34/38). Among completers, almost every participant rated the quality of the video (35/38, 92%) and audio (34/38, 89%) files as rather good or good.

The app-based support service can also be considered satisfactory (see the aforementioned results).

## Discussion

### Principal Findings

This study investigated the acceptability and feasibility of the implementation of an app-based support service in the daily lives of patients with severe mental illnesses in psychiatric outpatient care in addition to individual and group therapy sessions. The implementation was successful, as shown by the app’s feasibility, high user satisfaction, and users’ overall mental health status outcomes.

First, most participants used the app as instructed, which is a notable finding despite previous research suggesting that mental illnesses may be a common cause of noncompliance [46-48] and adherence rates for patients with mental disorders being shown to be rather low [49-51]. Most participants in the final sample used the app as indicated once per week, a high number of participants even used the app daily or more than twice a week despite their mental instability. The high level of user-friendliness, with almost all participants evaluating the app as rather or very easy to use, may explain this [52,53]. After the introduction meeting and joint app installation, all participants in the final sample were able to use the app independently. The handing out of the installation guide and app manual may have contributed to the problem-free use of the app, as did the seemingly intuitive app content. Almost every participant rated the quality of the video and audio files as rather good or good. The high-quality content of the video and audio files may have led to increased acceptance. These results provide significant support for the hypothesis that such an intervention is both feasible and acceptable in a psychiatric outpatient setting.

Various features of the app were used very frequently. These included, above all, the EMA-like approach. All 3 questionnaires were answered approximately equally often; this may be due to the reminders via push notifications as previous research has shown that push notification delivery is linked to higher mHealth app engagement [54] but could also be potentially due to the input via slider being simple and fast and, therefore, seemingly user-friendly even though this approach can lead to longer reaction times [55]. This supports our hypothesis of feasibility, especially the app-based EMA-like approach, as shown in a previous paper [56]. Activity button use was moderate, yet most participants felt that the button encouraged the performance of an activity, which can be considered a success as patients with severe mental disorders can experience a lack of daily structure or motivation to perform an activity but benefit from increased activity, which can lead to symptom stabilization [57-60]. Nevertheless, it was surprising that, despite the overall high use of the app in general, the individual relaxation modules were used little. Reminders to use the app via push notifications, as with the daily questionnaires, as well as deeper involvement of the target group in the development of app content and the option to personalize the app to meet individual requirements, could contribute to increased use, as previous studies have shown [61,62]. More participants from the full cohort did not use any modules at all, and fewer participants tried out different modules, whereas more participants from the completer group tried different modules and fewer participants did not use the modules at all. Overall, also in terms of EMA-like questionnaire responses and activity button use, the use of the app components was higher in the completer group. On the one hand, this can be explained by the longer period of use of the completers, but on the other hand, there may also be other reasons that might be found by further regarding the reason for a dropout. A subsequent work by Kaufmann et al [23] provides more details.

Second, the high satisfaction scores according to the mean sum scores on the ZUF-8 show that the intervention was satisfactory for participants and was able to support them to a high degree; the assessment of the ease of use emphasizes this, as well as the fact that most participants would use apps as support again and would recommend this type of support to a friend in a similar situation. This is also emphasized by the good final evaluation by means of a school grade, as well as the fact that most participants rated the app’s overall usability as rather satisfactory or satisfactory. These findings suggest that participants received the support that was supposed to be provided and that, in general, people with severe mental disorders can benefit from app-based support interventions as they can be highly satisfactory [63].

As the study was conducted in a psychiatric outpatient setting where individuals with rather severe illnesses were treated, an additional overall goal was to achieve stability of symptoms in patients who are severely ill. Even though the study population showed moderate to severe symptoms, app use was found to be high. Not only were the participants’ depressive symptoms successfully stabilized, but it was also possible to achieve a significant reduction in depressive symptoms. While the effect size for this was small, there was a slight decrease in the PHQ-9 score. The frequency of performing the SOP also decreased in total numbers, meaning that the number of participants with suicidal and self-injurious thoughts decreased. Even patients with severe mental illnesses were able to use and benefit from the app over a period of 4 weeks despite their pronounced symptomatology.

QOL remained stable at a medium level and even showed significant improvement in the domain environment. The nonsignificant improvements in the 3 domains of physical health, psychological health, and social relations and the significant improvement in the environment domain can be considered a success. Despite the ongoing high levels of psychological stress due to the pandemic that the study revealed, there was no significant decrease in QOL across domains, although previous studies have shown that overall QOL declined in both the general population and in patients with severe mental illnesses during the COVID-19 pandemic [64-67]. This app could have contributed to support patients and stabilize their mental health. However, it is also important to note that a study period of 4 weeks only covers a small section in view of the pandemic duration of almost 3 years and it is not feasible to infer that the stabilization of and improvements in participants’ mental health observed in this study were only the result of the app intervention due to the noncontrolled and nonrandomized study design and potential impacts of other concurrent continuing treatments.

On average, treatment credibility and expectancy were medium-high but showed a significant decrease over the study period. This may be explained by the fact that the app itself was as frequently used as intended but the CEQ refers to whether people believe that the app will reduce symptoms and that the expected effect of the app will be efficient. With this in mind, it does not seem unreasonable that the participants did not believe that using the app would significantly improve their condition as this was not the primary aim of the study; it was a feasibility study and not an efficacy study. Therefore, the study’s conclusion that this app is feasible and acceptable was not affected. However, this result may indicate that, in the future, psychotherapeutic content and methods and not exclusively relaxation methods should be at the forefront of the app. According to our findings, participants who had higher treatment expectancy scores after the intervention had significantly lower PHQ-9 sum scores at the posttreatment time point, suggesting a significant correlation between high treatment expectancy and improvement in posttreatment depressive symptoms. Regarding effect sizes, these results showed moderate effects. These results are consistent with those of other studies that have demonstrated a strong correlation between treatment expectancy and credibility and outcome scores in several research fields, including physical and mental health [68-72].

The extension of this form of app-based support to other subsets of psychiatric patient care should be considered. Some patients have difficulties adhering to their medication plan and continuously taking the prescribed medication. The app could be extended to this area, and compliance could be increased by means of a reminder function and a medication diary [73], as well as through the use of rewards in the form of awards and progress bars for gamification as this seems to be helpful to increase compliance, although this area requires further research [28]. Promising results have already been achieved via mobile apps in other disciplines in terms of increased compliance with medication adherence, as recently confirmed by a meta-study [74].

In summary, for most patients, supplementary app-based therapy offerings appear to be a promising therapeutic choice. Apps can be a promising way to support patients in their everyday lives, not only during the pandemic but also now in the postpandemic period, for several reasons. First, such an app can also be a way of monitoring the patients’ progress both for the users themselves and for the physician or therapist in the outpatient department. Second, mHealth apps represent a possibility to close a gap in care and provide support for actual psychotherapy [75]. In Germany in 2018, depending on the federal state, it took an average of 5.7 weeks to receive an initial consultation with a psychotherapist and up to 19.9 weeks to receive psychotherapy. The average waiting time in major German cities was approximately 4 months, whereas it was 5 to 6 months outside of major cities [76].

### Strengths and Limitations

The strengths of this study include not only the fact that our findings indicate that the implementation of an app-based support service is generally feasible and acceptable for people undergoing outpatient treatment but also that it could be demonstrated that such apps may be useful even for patients with severe mental illnesses. This was demonstrated not only via quantitative assessment but also via qualitative data from open questions administered through a feedback function at the postintervention time point on the app and an open telephone interview at the postintervention time point. This provided extended insights into patients’ experience. As user involvement is necessary to best adapt an app to users’ needs [22], the focus group meeting before app development can also be considered a strength of this study.

Limitations include the fact that this was not a randomized controlled study, and as a result, it cannot be used to verify the efficacy of app-based support services. However, other studies have already proven the effectiveness of various apps as support for ongoing therapy in the mental health sector and beyond [77-79]. The range of study participants was deliberately broad, but male participants were underrepresented. In addition, this study did not examine whether an app-based support service may be more useful for a particular group of patients with a particular diagnosis than for another. While the study covered a 4-week period, discussing strategies for maintaining user engagement over longer periods could be insightful. The dropout rate from T1 (during the intervention) to T2 (postintervention time point) was relatively high. The data that were ultimately used for analysis came from only 67% (38/57) of the participants who had originally started using the app, so these data do not represent all participants. Due to this, the analyses might be biased in that they focus on the outcomes of those who completed the study. Nevertheless, it should be mentioned that the reasons for dropout are being analyzed in a separate work, meaning that a special focus was placed on this.

### Conclusions

App-based support services are feasible, usable, and easy to implement in clinical routine care and can serve as a support to face-to-face individual and group therapy for patients with severe mental illnesses. They are also highly satisfactory and can not only stabilize depressive symptoms but also potentially lead to a decrease in symptoms. Thus, they may be a useful tool for future e–mental health services provided as part of standard clinical care.

## Abbreviations

CEQ: Credibility/Expectancy Questionnaire
EMA: ecological momentary assessment
mHealth: mobile health
PHQ-9: Patient Health Questionnaire–9
QOL: quality of life
SOP: standard operating procedure
WHOQOL-BREF: World Health Organization Quality of Life assessment
ZUF-8: German version of the Client Satisfaction Questionnaire–8

## References

[1] Lecomte T, Potvin S, Corbière M, Guay S, Samson C, Cloutier B, Francoeur A, Pennou A, Khazaal Y (2020). Mobile apps for mental health issues: meta-review of meta-analyses. JMIR Mhealth Uhealth.

[2] Bruhns A, Lüdtke T, Moritz S, Bücker L (2021). A mobile-based intervention to increase self-esteem in students with depressive symptoms: randomized controlled trial. JMIR Mhealth Uhealth.

[3] Whitehead L, Seaton P (2016). The effectiveness of self-management mobile phone and tablet apps in long-term condition management: a systematic review. J Med Internet Res.

[4] Mistler LA, Ben-Zeev D, Carpenter-Song E, Brunette MF, Friedman MJ (2017). Mobile mindfulness intervention on an acute psychiatric unit: feasibility and acceptability study. JMIR Ment Health.

[5] Wang K, Varma DS, Prosperi M (2018). A systematic review of the effectiveness of mobile apps for monitoring and management of mental health symptoms or disorders. J Psychiatr Res.

[6] (2016). mHealth app developer economics 2016. Research 2 Guidance.

[7] (2015). Patient adoption of mHealth: use, evidence and remaining barriers to mainstream acceptance. IMS Institute for Healthcare Informatics.

[8] (2021). Digital health trends 2021: innovation, evidence, regulation, and adoption. IQVIA.

[9] Webelhorst C, Jepsen L, Rummel-Kluge C (2020). Utilization of e-mental-health and online self-management interventions of patients with mental disorders-a cross-sectional analysis. PLoS One.

[10] Kalckreuth S, Trefflich F, Rummel-Kluge C (2014). Mental health related internet use among psychiatric patients: a cross-sectional analysis. BMC Psychiatry.

[11] Rauschenberg C, Schick A, Hirjak D, Seidler A, Paetzold I, Apfelbacher C, Riedel-Heller SG, Reininghaus U (2021). Evidence synthesis of digital interventions to mitigate the negative impact of the COVID-19 pandemic on public mental health: rapid meta-review. J Med Internet Res.

[12] Kim J, Marcusson-Clavertz D, Yoshiuchi K, Smyth JM (2019). Potential benefits of integrating ecological momentary assessment data into mHealth care systems. Biopsychosoc Med.

[13] Shiffman S, Stone AA, Hufford MR (2008). Ecological momentary assessment. Annu Rev Clin Psychol.

[14] Miralles I, Granell C, Díaz-Sanahuja L, Van Woensel W, Bretón-López J, Mira A, Castilla D, Casteleyn S (2020). Smartphone apps for the treatment of mental disorders: systematic review. JMIR Mhealth Uhealth.

[15] Graham AK, Greene CJ, Kwasny MJ, Kaiser SM, Lieponis P, Powell T, Mohr DC (2020). Coached mobile app platform for the treatment of depression and anxiety among primary care patients: a randomized clinical trial. JAMA Psychiatry.

[16] Mohr DC, Tomasino KN, Lattie EG, Palac HL, Kwasny MJ, Weingardt K, Karr CJ, Kaiser SM, Rossom RC, Bardsley LR, Caccamo L, Stiles-Shields C, Schueller SM (2017). IntelliCare: an eclectic, skills-based app suite for the treatment of depression and anxiety. J Med Internet Res.

[17] Roncero M, Belloch A, Doron G (2018). A novel approach to challenging OCD related beliefs using a mobile-app: an exploratory study. J Behav Ther Exp Psychiatry.

[18] Regier DA, Farmer M E, Rae D S, Locke B Z, Keith S J, Judd L L, Goodwin F K (1990). Comorbidity of mental disorders with alcohol and other drug abuse. Results from the Epidemiologic Catchment Area (ECA) Study. JAMA.

[19] Kessler RC, Chiu WT, Demler O, Merikangas KR, Walters EE (2005). Prevalence, severity, and comorbidity of 12-month DSM-IV disorders in the National Comorbidity Survey Replication. Arch Gen Psychiatry.

[20] McQuaid RJ (2021). Transdiagnostic biomarker approaches to mental health disorders: consideration of symptom complexity, comorbidity and context. Brain Behav Immun Health.

[21] LeBouthillier DM, Asmundson GJ (2017). The efficacy of aerobic exercise and resistance training as transdiagnostic interventions for anxiety-related disorders and constructs: a randomized controlled trial. J Anxiety Disord.

[22] Mohr DC, Riper H, Schueller SM (2018). A solution-focused research approach to achieve an implementable revolution in digital mental health. JAMA Psychiatry.

[23] Kaufmann L, Baldofski S, Golsong K, Kohls E, Rummel-Kluge C Reasons for nonparticipation and dropout in a longitudinal study of an app–based support service among adult patients in a psychiatric outpatient setting during the COVID-19 pandemic: cross-sectional study. JMIR Preprints.

[24] Yim SJ, Lui LM, Lee Y, Rosenblat JD, Ragguett RM, Park C, Subramaniapillai M, Cao B, Zhou A, Rong C, Lin K, Ho RC, Coles AS, Majeed A, Wong ER, Phan L, Nasri F, McIntyre RS (2020). The utility of smartphone-based, ecological momentary assessment for depressive symptoms. J Affect Disord.

[25] Manger S (2019). Lifestyle interventions for mental health. Aust J Gen Pract.

[26] Rosenbaum S, Tiedemann A, Sherrington C, Curtis J, Ward PB (2014). Physical activity interventions for people with mental illness: a systematic review and meta-analysis. J Clin Psychiatry.

[27] Mestdagh M, Verdonck S, Piot M, Niemeijer K, Kilani G, Tuerlinckx F, Kuppens P, Dejonckheere E (2023). m-Path: an easy-to-use and highly tailorable platform for ecological momentary assessment and intervention in behavioral research and clinical practice. Front Digit Health.

[28] Tran S, Smith L, El-Den S, Carter S (2022). The use of gamification and incentives in mobile health apps to improve medication adherence: scoping review. JMIR Mhealth Uhealth.

[29] Schmidt J, Lamprecht F, Wittmann WW (1989). [Satisfaction with inpatient management. Development of a questionnaire and initial validity studies]. Psychother Psychosom Med Psychol.

[30] Attkisson CC, Zwick R (1982). The client satisfaction questionnaire: psychometric properties and correlations with service utilization and psychotherapy outcome. Eval Program Plan.

[31] Nuij C, van Ballegooijen W, de Beurs D, de Winter RF, Gilissen R, O'Connor RC, Smit JH, Kerkhof A, Riper H (2022). The feasibility of using smartphone apps as treatment components for depressed suicidal outpatients. Front Psychiatry.

[32] Juniar D, van Ballegooijen W, Schulte M, van Schaik A, Passchier J, Heber E, Lehr D, Sadarjoen SS, Riper H (2022). A web-based stress management intervention for university students in Indonesia (Rileks): feasibility study using a pretest-posttest design. JMIR Form Res.

[33] Humbert A, Kohls E, Baldofski S, Epple C, Rummel-Kluge C (2023). Acceptability, feasibility, and user satisfaction of a virtual reality relaxation intervention in a psychiatric outpatient setting during the COVID-19 pandemic. Front Psychiatry.

[34] Kroenke K, Spitzer RL, Williams JB (2001). The PHQ-9: validity of a brief depression severity measure. J Gen Intern Med.

[35] Costantini L, Pasquarella C, Odone A, Colucci ME, Costanza A, Serafini G, Aguglia A, Belvederi Murri M, Brakoulias V, Amore M, Ghaemi SN, Amerio A (2021). Screening for depression in primary care with Patient Health Questionnaire-9 (PHQ-9): a systematic review. J Affect Disord.

[36] Levis B, Benedetti A, Thombs BD, DEPRESsion Screening Data (DEPRESSD) Collaboration (2019). Accuracy of Patient Health Questionnaire-9 (PHQ-9) for screening to detect major depression: individual participant data meta-analysis. BMJ.

[37] Bush NE, Skopp N, Smolenski D, Crumpton R, Fairall J (2013). Behavioral screening measures delivered with a smartphone app: psychometric properties and user preference. J Nerv Ment Dis.

[38] von Angermeyer MC, Kilian R, Matschinger H (2000). WHOQOL-100 und WHOQOL-BREF Handbuch für die deutschsprachigen Versionen der WHO Instrumente zur Erfassung von Lebensqualität.

[39] Skevington SM, Lotfy M, O'Connell KA, WHOQOL Group (2004). The World Health Organization's WHOQOL-BREF quality of life assessment: psychometric properties and results of the international field trial. A report from the WHOQOL group. Qual Life Res.

[40] Devilly GJ, Borkovec TD (2000). Psychometric properties of the credibility/expectancy questionnaire. J Behav Ther Exp Psychiatry.

[41] Scholl J, Kohls E, Görges F, Steinbrecher M, Baldofski S, Moessner M, Rummel-Kluge C (2021). Acceptability and feasibility of the transfer of face-to-face group therapy to online group chats in a psychiatric outpatient setting during the COVID-19 pandemic: longitudinal observational study. JMIR Form Res.

[42] (2022). Diese Öffnungsschritte haben Bund und Länder beschlossen. Die Bundesregierung.

[43] Sekhon M, Cartwright M, Francis JJ (2017). Acceptability of healthcare interventions: an overview of reviews and development of a theoretical framework. BMC Health Serv Res.

[44] Fisher P, McCarney R, Hasford C, Vickers A (2006). Evaluation of specific and non-specific effects in homeopathy: feasibility study for a randomised trial. Homeopathy.

[45] Hommel KA, Hente E, Herzer M, Ingerski LM, Denson LA (2013). Telehealth behavioral treatment for medication nonadherence: a pilot and feasibility study. Eur J Gastroenterol Hepatol.

[46] Bener A, Dafeeah EE, Salem MO (2013). A study of reasons of non-compliance of psychiatric treatment and patients' attitudes towards illness and treatment in Qatar. Issues Ment Health Nurs.

[47] Qian J, Simoni-Wastila L, Rattinger GB, Zuckerman IH, Lehmann S, Wei YJ, Stuart B (2014). Association between depression and maintenance medication adherence among Medicare beneficiaries with chronic obstructive pulmonary disease. Int J Geriatr Psychiatry.

[48] Cramer JA, Rosenheck R (1998). Compliance with medication regimens for mental and physical disorders. Psychiatr Serv.

[49] Colom F, Vieta E (2002). Non-adherence in psychiatric disorders: misbehaviour or clinical feature?. Acta Psychiatr Scand.

[50] Taj R, Khan S (2005). A study of reasons of non-compliance to psychiatric treatment. J Ayub Med Coll Abbottabad.

[51] Taj F, Tanwir M, Aly Z, Khowajah AA, Tariq A, Syed FK, Waqar F, Shahzada K (2008). Factors associated with non-adherence among psychiatric patients at a tertiary care hospital, Karachi, Pakistan: a questionnaire based cross-sectional study. J Pak Med Assoc.

[52] Jakob R, Harperink S, Rudolf AM, Fleisch E, Haug S, Mair JL, Salamanca-Sanabria A, Kowatsch T (2022). Factors influencing adherence to mHealth apps for prevention or management of noncommunicable diseases: systematic review. J Med Internet Res.

[53] Wang C, Qi H (2021). Influencing factors of acceptance and use behavior of mobile health application users: systematic review. Healthcare (Basel).

[54] Bidargaddi N, Almirall D, Murphy S, Nahum-Shani I, Kovalcik M, Pituch T, Maaieh H, Strecher V (2018). To prompt or not to prompt? A microrandomized trial of time-varying push notifications to increase proximal engagement with a mobile health app. JMIR Mhealth Uhealth.

[55] Gummer T, Vogel V, Kunz T, Roßmann J (2019). Let’s put a smile on that scale: findings from three web survey experiments. Int J Mark Res.

[56] Burke L, Naylor G (2022). Smartphone app-based noncontact ecological momentary assessment with experienced and naïve older participants: feasibility study. JMIR Form Res.

[57] Zimmermann M, Chong AK, Vechiu C, Papa A (2020). Modifiable risk and protective factors for anxiety disorders among adults: a systematic review. Psychiatry Res.

[58] Pearce M, Garcia L, Abbas A, Strain T, Schuch FB, Golubic R, Kelly P, Khan S, Utukuri M, Laird Y, Mok A, Smith A, Tainio M, Brage S, Woodcock J (2022). Association between physical activity and risk of depression: a systematic review and meta-analysis. JAMA Psychiatry.

[59] Yang XH, Huang J, Zhu CY, Wang YF, Cheung EF, Chan RC, Xie GR (2014). Motivational deficits in effort-based decision making in individuals with subsyndromal depression, first-episode and remitted depression patients. Psychiatry Res.

[60] Jimenez DE, Thomas L, Bartels SJ (2019). The role of serious mental illness in motivation, participation and adoption of health behavior change among obese/sedentary Latino adults. Ethn Health.

[61] Gerner M, Vuillerme N, Aubourg T, Messner EM, Terhorst Y, Hörmann V, Ganzleben I, Schenker H, Schett G, Atreya R, Neurath MF, Knitza J, Orlemann T (2022). Review and analysis of german mobile apps for inflammatory bowel disease management using the mobile application rating scale: systematic search in app stores and content analysis. JMIR Mhealth Uhealth.

[62] Chan AH, Honey ML (2022). User perceptions of mobile digital apps for mental health: acceptability and usability - an integrative review. J Psychiatr Ment Health Nurs.

[63] Kidd SA, Feldcamp L, Adler A, Kaleis L, Wang W, Vichnevetski K, McKenzie K, Voineskos A (2019). Feasibility and outcomes of a multi-function mobile health approach for the schizophrenia spectrum: App4Independence (A4i). PLoS One.

[64] Tripoli G, Lo Duca S, Ferraro L, Zahid U, Mineo R, Seminerio F, Bruno A, Di Giorgio V, Maniaci G, Marrazzo G, Sartorio C, Scaglione A, La Barbera D, La Cascia C (2024). Lifestyles and quality of life of people with mental illness during the COVID-19 pandemic. Community Ment Health J.

[65] Hansel TC, Saltzman LY, Melton PA, Clark TL, Bordnick PS (2022). COVID-19 behavioral health and quality of life. Sci Rep.

[66] Dale R, Budimir S, Probst T, Humer E, Pieh C (2022). Quality of life during the COVID-19 pandemic in Austria. Front Psychol.

[67] Violato M, Pollard J, Lloyd A, Roope LS, Duch R, Becerra MF, Clarke PM (2023). The COVID-19 pandemic and health-related quality of life across 13 high- and low-middle-income countries: a cross-sectional analysis. PLoS Med.

[68] Srinivas P, Bodke K, Ofner S, Keith NR, Tu W, Clark DO (2019). Context-sensitive ecological momentary assessment: application of user-centered design for improving user satisfaction and engagement during self-report. JMIR Mhealth Uhealth.

[69] Smeets RJ, Beelen S, Goossens ME, Schouten EG, Knottnerus JA, Vlaeyen JW (2008). Treatment expectancy and credibility are associated with the outcome of both physical and cognitive-behavioral treatment in chronic low back pain. Clin J Pain.

[70] Haanstra TM, Tilbury C, Kamper SJ, Tordoir RL, Vliet Vlieland TP, Nelissen RG, Cuijpers P, de Vet HC, Dekker J, Knol DL, Ostelo RW (2015). Can optimism, pessimism, hope, treatment credibility and treatment expectancy be distinguished in patients undergoing total hip and total knee arthroplasty?. PLoS One.

[71] Groeneveld IF, Goossens PH, van Braak I, van der Pas S, Meesters JJ, Rambaran Mishre RD, Arwert HJ, Vliet Vlieland TP (2019). Patients' outcome expectations and their fulfilment in multidisciplinary stroke rehabilitation. Ann Phys Rehabil Med.

[72] Goossens ME, Vlaeyen JW, Hidding A, Kole-Snijders A, Evers SM (2005). Treatment expectancy affects the outcome of cognitive-behavioral interventions in chronic pain. Clin J Pain.

[73] Ng R, Carter SR, El-Den S (2020). The impact of mobile applications on medication adherence: a systematic review. Transl Behav Med.

[74] Al-Arkee S, Mason J, Lane DA, Fabritz L, Chua W, Haque MS, Jalal Z (2021). Mobile apps to improve medication adherence in cardiovascular disease: systematic review and meta-analysis. J Med Internet Res.

[75] Chandrashekar P (2018). Do mental health mobile apps work: evidence and recommendations for designing high-efficacy mental health mobile apps. Mhealth.

[76] (2018). Ein jahr nach der reform der psychotherapie‐richtlinie. Bundes Psychotherapeuten Kammer.

[77] Hur JW, Kim B, Park D, Choi SW (2018). A scenario-based cognitive behavioral therapy mobile app to reduce dysfunctional beliefs in individuals with depression: a randomized controlled trial. Telemed J E Health.

[78] Arean PA, Hallgren KA, Jordan JT, Gazzaley A, Atkins DC, Heagerty PJ, Anguera JA (2016). The use and effectiveness of mobile apps for depression: results from a fully remote clinical trial. J Med Internet Res.

[79] Astafeva D, Kolsanov A, Chaplygin S, Yashikhina A, Cumming P, Vlasov A, Syunyakov T, Smirnova D (2022). The efficacy of mobile phone-based interventions for the treatment of depression: a systematic meta-review of meta-analyses of randomized controlled trials. Psychiatr Danub.

